# Glial Cell AMPA Receptors in Nervous System Health, Injury and Disease

**DOI:** 10.3390/ijms20102450

**Published:** 2019-05-17

**Authors:** Maria Ceprian, Daniel Fulton

**Affiliations:** 1 Instituto de Investigación Sanitaria San Carlos (IdISSC), 28040 Madrid, Spain; mceprian@ucm.es; 2 Departamento de Bioquímica y Biología Molecular, CIBERNED, IRICYS. Facultad de Medicina, Universidad Complutense de Madrid, 28040 Madrid, Spain; 3 Neuroscience and Ophthalmology Research Group, Institute of Inflammation and Ageing, College of Medical and Dental Sciences, University of Birmingham, Edgbaston, Birmingham B15 2TT, UK

**Keywords:** glia, AMPA receptor, astrocyte, microglia, glutamate, excitotoxicity, inflammation, hypoxia, ischemia, multiple sclerosis, neurodegeneration

## Abstract

Glia form a central component of the nervous system whose varied activities sustain an environment that is optimised for healthy development and neuronal function. Alpha-amino-3-hydroxy-5-methyl-4-isoxazole (AMPA)-type glutamate receptors (AMPAR) are a central mediator of glutamatergic excitatory synaptic transmission, yet they are also expressed in a wide range of glial cells where they influence a variety of important cellular functions. AMPAR enable glial cells to sense the activity of neighbouring axons and synapses, and as such many aspects of glial cell development and function are influenced by the activity of neural circuits. However, these AMPAR also render glia sensitive to elevations of the extracellular concentration of glutamate, which are associated with a broad range of pathological conditions. Excessive activation of AMPAR under these conditions may induce excitotoxic injury in glial cells, and trigger pathophysiological responses threatening other neural cells and amplifying ongoing disease processes. The aim of this review is to gather information on AMPAR function from across the broad diversity of glial cells, identify their contribution to pathophysiological processes, and highlight new areas of research whose progress may increase our understanding of nervous system dysfunction and disease.

## 1. Introduction

Glutamatergic signaling through alpha-amino-3-hydroxy-5-methyl-4-isoxazole (AMPA) receptors (AMPAR) forms a major component of excitatory synaptic transmission in the central nervous system (CNS). However, glutamate release in the CNS is not exclusive to synaptic terminals, but also arises from unmyelinated axons [1] and non-neuronal glial cells [2] under both physiological and pathophysiological conditions. Glutamate is therefore present at varying concentrations in a number of extra-synaptic locations where it influences non-neuronal AMPAR, particularly those expressed by CNS glia cells, leading to influences on a range of critical functions. Importantly, glutamate release is enhanced under a number of pathological conditions [3,4,5] leading to concentrations of extracellular glutamate that trigger excitotoxic processes that threatens glial cell viability, and set in motion cellular and molecular processes that initiate and intensify pathophysiological conditions. In this review we provide an overview of AMPAR expression in glial cells of the CNS and peripheral nervous system (PNS), describing functions for these receptors in physiological conditions, highlighting their involvement in glial responses to pathophysiological conditions, and hypothesising on additional roles that glial AMPAR may perform in the context of nervous system injury and disease. The review will also indicate areas for future research, including cannabinoid AMPAR interactions, and AMPAR-stimulated transcriptional regulation, whose investigation promises to stimulate new knowledge on mechanisms regulating injury and disease processes, and identify new targets for CNS protection and repair.

### 1.1. Glia: A Brief Overview of Diversity in Form and Function

Glia are a diverse group of neural cells whose principle uniting features are a non-neuronal identity, and the performance of functions essential to the normal operation of the nervous system. Glia can be divided into two categories based on their developmental origins: macroglia derived from the ectoderm; and microglia derived from hemopoietic stem cells originating from the yolk sac during early embryonic development [6]. The principle CNS macroglia are the astrocytes and oligodendrocytes (OL). Astrocytes are a heterogenous group of cells that, in addition to a well-defined role in optimising extracellular conditions for neuronal function via the uptake of neurotransmitters and ions, may also contribute to the regulation of a number of other functions including synaptic function, cerebral blood flow and maintenance of the blood brain barrier [7]. OL are exclusively involved in myelin generation in the CNS [8]. However, it is now appreciated that myelination provides benefits to their neuronal targets that extend beyond the enhancement of axonal conduction velocities to encompass the provision of trophic and metabolic support [9], and potentially an involvement in information processing and and learning [10,11]. CNS macroglia also include NG2-glia, radial glia, and ependymal cells. NG2- glia are characterised as multi-process bearing NG2/PDGF receptor α expressing cells capable of generating OL and astrocytes (and possibly neurons, although this is the subject of intense debate [12]) in both the embryonic and postnatal CNS [13]. However, their extensive distribution throughout the adult CNS, and synaptic integration with neural circuity, suggest functions that extend beyond the role of a glial progenitor [13,14,15]. Radial glia are astrocyte-like neural progenitors that sequentially give rise to neurons, astrocytes and OL during embryonic development [16]. They are also considered to be the progenitors for the CNS ependymal cells (see below) [17]. Structurally, radial glia are polarised cells with a cell body located close to the ventricle, a short “endfoot” oriented towards the ventricle wall, and a longer process that extends to make contact with the pial surface [18]. This morphological arrangement provides a scaffold that guides newly born neurons as they migrate into the developing cerebral cortex [19]. Ependymal cells cover the brain’s ventricles and the central canal of the spinal cord. Examples of ependymal cells include the cerebral spinal fluid secreting choroid plexus epithelial cells [20], and thalamic tanycytes. Tanycytes are radial glia-like cells located in the walls of the 3rd and 4th ventricle, that exhibit a neuro- and gliogenic capacity, and through their function as glucosensors, are proposed to play a role in regulating feeding and energy balance [21]. Microglia, which are restricted to the CNS, present a dualistic identity, providing protective and modulatory influences under healthy conditions through the release of pro-survival trophic factors, and by tidying CNS tissues through the removal of dead cells and the pruning of excess synapses. However, when activated under pathological conditions they play a central role in the progression of CNS injury and disease states through the release of inflammatory and cytotoxic mediators [22]. Thus, microglia are essential to both the maintenance of a healthy CNS environment, and the pathophysiological processes that threaten it.

PNS macroglia include Schwann cells, satellite cells and enteric glia. Schwann cells exhibit two forms, the myelinating variety which form compact myelin sheaths on PNS axons, and non-myelinating forms whose interactions with multiple axons form the Remak fibres [23]. Under pathological conditions Schwann cells are capable of de-differentiating into an immature phenotype reported to exhibit pro-demyelination characteristics, while also expressing neurotrophic factors involved in neuronal survival and axonal regeneration [24]. Satellite glia interact with the soma of neurons in the sensory, sympathetic and parasympathetic ganglia where they are considered to play a similar role to astrocytes in the CNS. Finally, enteric glia are located in the ganglia of the gastrointestinal tract and are considered the most similar of PNS glia to astrocytes due to their multi-process morphology, including “end feet” connected to blood vessels, and their coupling via gap junctions to form a glial syncytium [25].

### 1.2. AMPAR

AMPAR are one of three types of ionotropic glutamate receptors, the others being the *N*-methyl-d-aspartate receptors (NMDAR), known for their involvement in synaptic plasticity [26], and the kainate receptors (KR). KR are frequently grouped with AMPAR due to their similar pharmacological properties (e.g., agonised by kainate, and antagonised by several of the same drugs). However, KR are formed from distinct protein subunits that confer unique receptor properties, such as slower inactivation kinetics, that distinguish them from AMPAR [27,28]. Although glutamate can influence glia via activation of NMDAR and KR, these actions have been reviewed elsewhere [29,30] and will not be covered in the present work. Regarding AMPAR function in neuronal circuits, AMPAR activation mediates the majority of fast excitatory synaptic communication in the CNS. AMPAR are heterotetrameric complexes containing various combinations of the pore forming GluA subunits 1–4 (GluA1–4) whose assembly produces cation permeable receptors with physiological properties that differ depending on their specific subunit composition [28]. AMPAR channel activation permits the influx of Na^+^, and varying levels of Ca^2+^, depending on the subunits present in the complex. Cation influx induces excitatory post synaptic currents (EPSCs) with rapid kinetics that are characterised by pronounced receptor desensitisation and influenced by subunit composition [31,32]. The inclusion of GluA2 limits the permeability of AMPAR to Ca^2+^ due to codon editing at the so-called Q/R site located within the pore forming region. The vast majority of GluA2 subunits undergo Q/R editing leading to the presence of a positively charged arginine residue within this region that inhibits the permeation of divalent ions [33,34]. In this way, AMPAR lacking GluA2 show greater Ca^2+^ permeability than complexes containing this subunit. Variation in AMPAR desensitisation of all GluA subunits arises through alternative splicing to produce Flip and Flop variants that differ in their kinetics of desensitisation [28]. Importantly, most Flip variants exhibit slower, less pronounced, desensitisation, thus a dominance of these subunits over the Flop variant in AMPAR would be expected to increase vulnerability to excitotoxic injury. This scenario may occur in amyotrophic lateral sclerosis (ALS) where motoneurons in the cervical ventral horn show a decrease in Flop variants, while Flip expression is sustained [35]. Diversity in AMPAR receptor function is also provided by a range of auxiliary proteins, most notably the transmembrane AMPAR regulatory proteins (TARPs), whose inclusion in the complex influences AMPAR pharmacology and kinetics, and regulates GluA subunit trafficking [36,37].

AMPAR function in the nervous system is not restricted to neuronal cells. Indeed many glial cells exhibit functional AMPAR whose activation regulates a range of important cellular activities including cell migration [38,39], morphological development and re-structuring [40,41] proliferation and differentiation [42,43], transcriptional regulation [44,45], survival [46], and the regulation of various ion channels [42,47,48]. Importantly, overactivation of AMPAR plays a key role in mediating cellular injury to neuronal and glial cells alike. These excitotoxic actions, which largely depend on the excessive influx of Ca^2+^, are described in the following section.

### 1.3. AMPAR Involvement in Cellular Injury

Glutamatergic excitotoxic injury is defined by the overactivation of glutamate receptors due to high or prolonged glutamate exposure. Cell death induced by excitotoxicity is an important feature in either acute or neurodegenerative pathologies [4,49]. After an ischemic stroke the increase of glutamate level directly correlates to the severity of the stroke, infarct volume and poorer functional outcome of patients [50,51]. That correlation has also been observed in different rodent stroke models [52]. Similar results were observed in the cerebrospinal fluid (CSF) or cerebral areas of patients after a traumatic brain injury [53,54]. Higher levels of glutamate have also been described in the CSF of infants correlating with the severity of hypoxic-ischemic (H-I) encephalopathy [55], and indeed levels of glutamate remain elevated for days in animal models of pre-term H-I injury [56,57,58]. Glutamate excitotoxicity is also an important event in chronic neurodegenerative diseases [49]. Both Parkinson’s and Alzheimer’s diseases are characterized by a dysregulation of glutamate homeostasis that may damage neurons [59,60,61], and both serum and CSF from patients with Multiple Sclerosis (MS) and Amyotrophic Lateral Sclerosis (ALS) show higher levels of glutamate [5,62,63].

Excessive levels of glutamate induce cell death by several complex mechanisms that have been previously reviewed [49,60,64]. Importantly, sustained activation of AMPAR, NMDAR and KR under these conditions produce a large increase in intracellular Ca^2+^ [65,66,67] that triggers various cellular injury processes including endoplasmic reticulum (ER) stress, mitochondrial dysfunction and the production of reactive oxygen and nitrogen species [59,66,68,69,70,71,72,73,74,75]. Ca^2+^ influx also induces the activation of calcium-dependent enzymes that promote cell damage. This situation is exemplified by calpain, whose activation by elevated Ca^2+^ levels promotes the internalization of plasma membrane Ca^2+^ ATPase leading to further dysregulation of intracellular Ca^2+^ and oxidative stress [68,76,77]. These outcomes are related to ER stress since this organelle is highly sensitive to disturbances in calcium homeostasis and the reactive species that ensue under these conditions. Therefore, the ER is highly sensitive to glutamate insults and cytosolic Ca^2+^ elevations [68,69,71,78,79]. In addition to these actions, calpain may actively promote ER stress via the modulation of ryanodine receptor and the sarco/endoplasmic reticulum Ca^2+^-ATPase (SERCA) [71,80,81,82]. Here, alterations in ryanodine receptors and the SERCA produce further dysregulation of the Ca^2+^ balance providing an additional stimulus to the production of reactive oxygen species, which, together with reactive nitrogen species that also arise due to enhanced Ca^2+^ levels, promote oxidative stress [64,68,69,72,77,80,83,84]. Ca^2+^ accumulation and nitrogen oxide disrupt the electron transport chain in mitochondria producing additional reactive oxygen species and mitochondria dysfunction [64,68,77]. Indeed, glutamate induction of both mitochondria failure and ER stress are critical factors in excitotoxic cell death [59,64,68,69,74,77]. Glutamate insult further potentiates oxidative stress by decreasing glutathione and superoxide dismutase activity, and promoting NADPH oxidase activity, eventually leading to lipid peroxidation, protein nitrosylation and cell death [72,85]. In addition, glutamate excitotoxicity also increases Ca^2+^ uptake by mitochondria, leading to the opening of mitochondrial permeability transition pores, and mitochondria fragmentation [66,74]. The high levels of Ca^2+^ uptake by mitochondria have also been related to the inhibition of mitochondrial respiration and the release of cytochrome c, thus triggering apoptosis [75,86,87]. Glutamate excitotoxicity also increases the expression of pro-apoptotic Bak protein, while also decreasing anti-apoptotic Bcl-2 protein levels [71], and altering the transcription and function of the nuclear factor Y (NF-Y) complex [45], a transcription factor closely linked to the control of apoptotic cell death [88] (see Section 8).

### 1.4. AMPAR in Glial Cells

The vast majority of research into glial cells has focussed on the astrocytes, oligodendrocytes and microglia. Much is therefore known regarding the expression and function of AMPAR in these CNS glia, while knowledge on this topic in other glial cells, particularly those of the autonomic system and the ependymal tissues is limited. Consequently this review will largely focus on those glia where functional AMPAR have been most thoroughly explored, although we will attempt to summarise the information that is available on other glial subtypes where possible. The following sections provide a review of each glial cell type, considering AMPAR expression, describing the known functions for these receptors in physiological and pathophysiological conditions, and highlighting emergent actions that stimulation of these AMPAR may evoke in the context of nervous system injury and disease. The major findings on AMPAR receptor expression and function in each glial cell type are summarised in Table 1, Table 2, Table 3 and Table 4.

## 2. Astrocytes

Astrocytes are the most numerous glial cells in the CNS. They are distributed throughout both the grey and white matter where they perform a myriad of tasks that serve to maintain neuronal function. Astrocytes make extensive contacts with synapses and nodes of Ranvier that enable them to regulate neuronal environments. They achieve this through the re-uptake of transmitters, and the buffering of extracellular ions, which together help to provide an extracellular environment that is optimised for efficient axonal and synaptic activity. In addition to their connections to neuronal compartments, astrocytes extend processes that terminate on cerebral vasculature through which they are involved in the formation of the blood brain barrier [89], and in mediating neurovascular coupling to maintain the supply of energy to the brain [90]. Astrocytes also have important functions in guiding CNS development, being involved in both the regulation of myelination [91] and synaptogenesis [92]. Astrocytes are equipped with a diverse array of neurotransmitter receptors that enable them to monitor neuronal activity, and which may provide the capacity to generate feedback signals via “glio transmitters” whose actions on adjacent neuronal synapses may involve the regulation of basal transmission and the regulation of synaptic plasticity [93] (also see [94]). Many of these neurotransmitter actions, including those stemming from glutamate, are associated with the activation of metabotropic receptors [95]. In contrast, less is known regarding the influence of AMPAR in astrocytes. The following sections will review the available literature and discuss the potential functions of astrocyte AMPAR in physiological and pathophysiological conditions. A summary of these findings is presented in Table 1.

### 2.1. Expression and Functional Properties of AMPAR in Astrocytes

Whole-cell voltage-clamp recordings show that astrocytes in most CNS regions exhibit functional AMPAR [96,103,104,105]. The exception to this rule appears to be the hippocampus where AMPAR-mediated currents are absent from astrocytes exhibiting high levels of GFAP-GFP transgene expression (cells expressing low GFAP-GFP transgene are now recognised as oligodendrocyte progenitors) [102]. The subunit composition of astrocyte AMPAR differs from region to region leading to variability in their permeability to Ca^2+^. Bergmann glia in the cerebellum exhibit strong expression of transcripts for GluA1 and GluA4, and lower levels of GluA2, leading to AMPAR with a high level of Ca^2+^ permeability [40,96]. Similarly, astrocytes in the olfactory bulb (OB) express significant levels of GluA1 and 4 protein, and low levels of GluA2 [103]. Although AMPA stimulation evokes a measurable influx of Ca^2+^ in these cells, AMPAR-mediated currents in these astrocytes are not fully blocked by specific blockade of Ca^2+^ permeable AMPAR, thus OB astrocytes appear to express a mix of Ca^2+^ permeable and impermeable receptors [103]. In other CNS regions, such as the cortex, GluA2 represents a dominant AMPAR subunit in astrocytes, with GluA1 and 4 transcripts being present at considerably lower levels [100]. As expected, AMPAR stimulation fails to elicit Ca^2+^ influx in cortical astrocytes unless their desensitisation is blocked by the application of cyclothiazide (CTZ) [101] indicating a low permeability to Ca^2+^. In the spinal cord immunohistochemical analysis reveals GluA2, 3 and 4 protein on GFAP^+^ astrocytes [106], while astrocytes in the thalamus exhibit a low level of Ca^2+^ permeability, potentially due to a dominant expression of GluA2, as revealed by the partial sensitivity of their AMPAR currents to pharmacological agents that selectively block GluA2 lacking receptors [104].

### 2.2. Astrocyte AMPAR Functions Under Physiological Conditions

**Figure 1:**
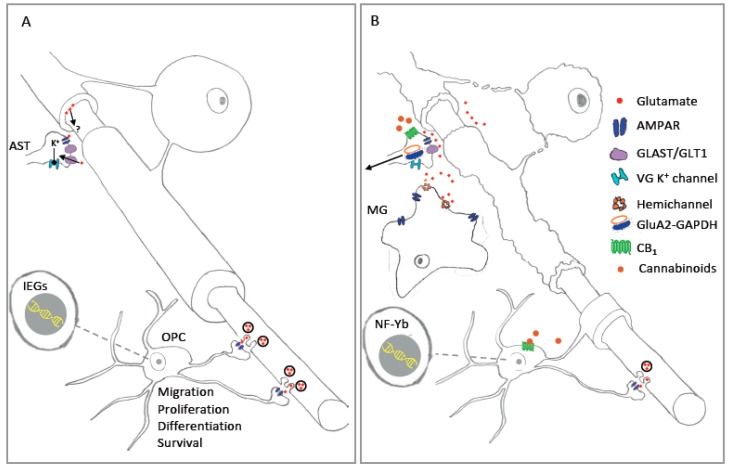
AMPAR functions for CNS white matter glia in health and disease. (**A**) Illustration depicting the interaction of glial elements in CNS white matter. Astrocytes (AST) and oligodendrocyte progenitors (OPC) extend processes that make contact with unmyelinated axons and the nodes of Ranvier on myelinated axons. These glial processes contain functional AMPAR since white matter astrocytes exhibit AMPAR-mediated Ca^2+^ signals, and OPC display AMPAR-mediated synaptic currents. Both unmyelinated and myelinated axons release glutamate via vesicular mechanisms. Glutamate released at unmyelinated axons drives synaptic input on OPC, but it is unclear whether glutamate released at internodal sites diffuses in concentrations sufficient to activate glial AMPAR at nodes of Ranvier. The functional consequences of astrocyte AMPAR in white matter remains unknown but may include a role in the regulation of outward K^+^ currents. AMPAR activation influences multiple functions in OPC including migration, proliferation, differentiation and survival. In addition, AMPAR activation in OPC influences events in the nucleus including the induction of immediate early genes involved in cellular growth. Note, OPC actions depicted also occur in CNS grey matter. (**B**) Depiction of excitotoxic events and glial cell injury in CNS white matter. Excitotoxic and in inflammatory conditions involve an increase in extracellular glutamate levels that damage OL and myelin internodes. Glutamate is released from damaged axons and from gap-junction hemichannels on activated microglia. Excessive astrocyte AMPAR activation may aggravate excitotoxic conditions via the downregulation of astrocyte GLAST. Myelin can be restored by OPC recruited to demyelinated axons. GluA2-GAPDH complexes formed in astrocytes at inflammatory demyelinating lesions may undergo nuclear translocation leading to the initiation of disease processes. Myelin repair involves the recruitment of OPC to demyelinated axons where they establish AMPAR-mediated synaptic connections. Axon-OPC synapses may play a role in guiding OPC to target axons, and in controlling their differentiation into myelinating OL. The modulation of the Endocannabinoid System is able to prevent several of these pathogenic pathways. Either the increase of endocannabinoid tone, or the direct agonism of CB_1_ receptors, reduces cytosolic Ca^2+^ influx in the oligodendrocyte after an AMPA stimulus. Similarly, increased AEA tone prevents GLAST and GLT-1 downregulation in a mechanism that involves at least CB_1_ receptor, and AEA tone increase or CB_1_/ CB_2_ agonism potentiates GLAST and GLT-1 expression in mouse models of MS. Note, other cytotoxic mediators involved in inflammatory demyelination, such as cytokines and complement cascade components are not shown for clarity.

**Figure 2:**
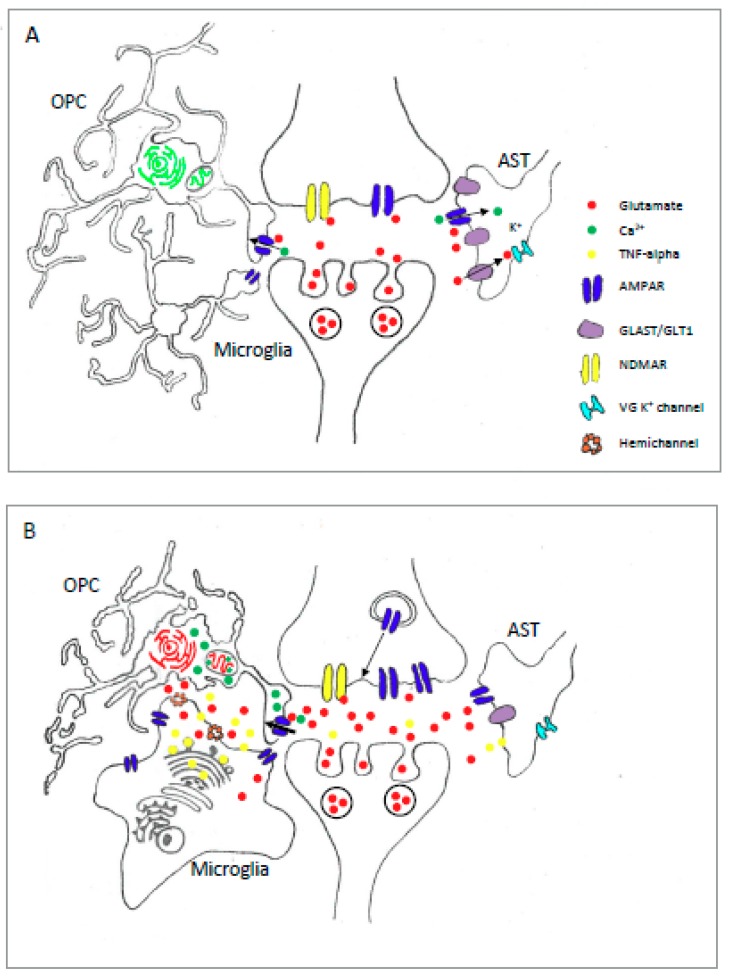
AMPAR functions for peri-synaptic CNS glia in health and disease. (**A**) In the healthy CNS astrocyte (AST) and OPC/NG2-glia processes exhibit physical and functional contacts with neuronal synapses. Ca^2+^ permeable AMPAR on Bergmann glia sustain the physical interaction between glial processes and neuronal synapses allowing efficient clearance of glutamate from Purkinje cell synapses. Activation of astrocyte AMPAR also induces a blockade of outward K^+^ currents that may support neuronal function during sustained periods of neuronal activity. OPC/NG2-glia in developing and adult CNS tissues exhibit Ca^2+^ permeable AMPAR and receive AMPAR-mediated synaptic input that may regulate their migration, maturation and survival. In the adult CNS NG2-glia continue to receive synaptic input, the functions of which remain unclear. The role of microglial AMPAR in the healthy CNS remain unknown although in vitro data suggest a role in chemotaxis [117]. (**B**) Glial cell AMPAR amplify pathological conditions and mediate glial cell injury in the CNS. Stimulation of AMPAR under excitotoxic conditions induces the release of glutamate from activated microglia via gap-junction hemichannels. AMPAR activation under hypoxic conditions also stimulates the upregulation of microglial AMPAR leading to an imbalance in anti- and pro-inflammatory cytokine release characterised by enhanced release of TNF-α. TNF-α released from activated microglia may intensify excitotoxic conditions by inducing the downregulation of astrocytic GLT1. Similarly, direct stimulation of astrocyte AMPAR may worsen excitotoxic conditions by inducing the downregulation of GLAST. TNF-α also increases the vulnerability of neurons to excitotoxicity by stimulating increased surface trafficking of AMPAR. Excessive AMPAR activation induces direct injury to OPC via numerous mechanisms including oxidative and ER stress and mitochondrial dysfunction (depicted by red organelles). In addition, excitotoxic stimulation of OPC AMPAR alters the function of transcription factor complex NF-Y leading to alterations in the expression of Ca^2+^ permeable GluA4 subunits and the regulation of genes involved in apoptosis.

Despite the abundance of studies exploring the expression of AMPAR in astrocytes (Section 2.2), relatively few physiological functions are ascribed to these receptors. In vitro studies using primary cultures of cortical astrocytes show that Na^+^ influx due to AMPAR activation produces a blockade of outward K^+^ currents through both A-type and delayed rectifier channels [48]. AMPAR activation in cultured Bergman glia produces similar effects on K^+^ currents activity [47,97]. Although this mechanism has not been confirmed in situ, it has been proposed to act as a mechanism for limiting elevations in extracellular [K^+^] during periods of excessive neuronal activity [48]. Astrocyte AMPAR are likely to be situated in appropriate locations to fulfil this role since astrocyte processes make extensive contact with synapses [92] and nodes of Ranvier throughout the CNS [107,108] (Figure 1A and Figure 2A). Glutamate has well documented effects on the function of peri-synaptic astrocytes [95], thus AMPAR-mediated effects on outward K^+^ currents seems a possibility. With regard to peri-nodal astrocytes, internodal axonal segments are known to release glutamate in an activity-dependent manner [109], yet it is unclear whether glutamate released in this way diffuses in sufficient concentrations to stimulate glial receptors located at nodes (Figure 1A). OL myelin). To do so it must cross the paranodal axoglial junctions (PAJ) that separate the internodal periaxonal and paranodal spaces. The PAJ has in fact been proposed as a route for the traffic of small molecules such as glucose [110], thus it is conceivable that glutamate may also diffuse via this structure to reach glial AMPAR located in peri-nodal spaces. Resolution of these questions could be achieved through the application of a genetically encoded glutamate sensing molecule, for example the intensity-based glutamate sensing fluorescent reporter (iGluSnFR) [111], which if targeted to astrocytes may be useful for revealing perinodal glutamate release. Alternatively, two-photon imaging may be used to analyse AMPAR-mediated Ca^2+^ influx at peri-nodal astrocyte process [112]. Studies of this nature could have wide-reaching impact given the important role astrocytes play in the regulation of myelination [91], and the influence that neuronal activity exerts in guiding oligodendrocyte differentiation and myelination [113]. Additionally, glutamate exerts a multitude of action in astrocyte via metabotropic glutamate receptors [95], thus evidence in support of an axonal-astrocyte glutamatergic signaling pathway could have significance for our understanding of white matter development that extends beyond the influence of AMPAR.

In contrast to the hypothetical functions of white matter astrocytes discussed above, AMPAR located on the processes of astrocytes in the molecular layer of the cerebellum perform an established function in the regulation of excitatory synapses. Here, Ca^2+^ influx through Ca^2+^-permeable AMPAR located on Bergman glial processes is linked to the regulation of glutamatergic synapses on Purkinje cell dendrites [40]. These synapses are enfolded by Bergman glia processes [114] whose expression of the glutamate transporter GLAST helps define the kinetics of AMPAR-mediated currents at Purkinje cells synapses. The function of these Ca^2+^-permeable AMPAR, which lack GluA2, are revealed by experiments in which the Ca^2+^ permeability of these AMPAR is reduced by exogenous expression of GluA2. Forced GluA2 expression leads to a retraction of the glial processes away from synapses, and an increase in the duration of Purkinje cell glutamatergic synaptic currents. These changes likely reflect alterations in the re-uptake of glutamate by Bergmann glial glutamate transporters located on the retracted processes [40]. Evidence for the significance of these synaptic effects are apparent in observations from transgenic mice with astrocyte-targeted deletions of GluA1 and 4. Here, changes in glial-neuron interactions and synaptic function are correlated with alterations in fine motor control that are consistent with the disturbances in Purkinje cell synaptic function [98].

### 2.3. Astrocyte AMPAR in Pathology

Astrocyte AMPAR activation has not been directly implicated in CNS injury or disease. Indeed, in contrast to cells in the OL lineage (Section 3.2), astrocytes do not appear to be vulnerable to pathological conditions associated with glutamate mediated excitotoxicity [106,115]. In fact, AMPAR mediated excitotoxicity is only observed in cultures of cortical astrocytes when AMPAR desensitisation is prevented by the application of CTZ [101]. Ca^2+^ imaging failed to reveal glutamate-mediated Ca^2+^ influx in these cultures suggesting that cortical astrocytes exhibit AMPAR with low Ca^2+^ permeability. These findings, and similar observations of excitotoxic resistance in hippocampal astrocytes [116], are supported by RNAseq data showing a dominance of GluA2 in both cortical and hippocampal astrocytes in situ [100]. Thus, low levels of AMPAR Ca^2+^ permeability may be associated with resistance to glutamate mediated injury. While excessive AMPAR activation does not appear to induce overt injury, it may trigger molecular alterations that could then trigger pathological conditions in the CNS. In this regard, prolonged stimulation of cultured Bergmann glia with AMPAR antagonists produces a downregulation in the expression of the glutamate transporter GLAST [99] (Figure 2B). Alterations in glial glutamate clearance due to a reduction in GLAST would be expected to disturb synaptic transmission, and could intensify excitotoxic conditions thus threatening more vulnerable neurons and OL. On this basis astrocyte AMPAR may represent a useful therapeutic target despite the apparent insensitivity to glutamate mediated injury of these glia.

Excitoxicity is a common feature of many CNS disease states including H-I injury, stroke, multiple sclerosis and neurodegenerative disorders including Alzheimer’s, Huntinton’s and Parkinson’s Disease [118]. Thus, CNS diseases of this type may provide valuable areas in which to search for subtle molecular alterations that could signal an involvement of astrocyte AMPAR in the initiation or propagation of the disease state. In this context, the emergence of RNAseq studies examining cell-specific gene expression in various disease models provides an opportunity to search for disease signatures that could foreshadow an involvement of glial AMPAR. The experimental autoimmune encephalomyelitis (EAE) model of inflammatory demyelination provides a promising model for this line of enquiry since AMPAR-mediated excitotoxicity has been linked to disease processes in this model [119,120,121]. In agreement with this, a recent astrocyte transcriptome analysis in the EAE model reveals a down-regulation of GluA4 in spinal cord astrocytes [122]. The consequence of this alteration in AMPAR expression are unclear. However, given the involvement of GluA4 in mediating Ca^2+^ influx, and the influence of astrocytes on myelin formation and viability [91], it is interesting to consider the detrimental effects that could arise due to an uncoupling of the axon-astrocytes interaction under inflammatory excitotoxic conditions. Although not direct implicating AMPAR activation, astrocytic GluA2 has also been linked to the pathogenesis of EAE. Recent work studying protein complexes containing GluA2 and glyceraldehyde 3-phosphate dehydrogenase (GAPDH), a key enzyme involved in glycolytic metabolism, have identified an increase in the presence of this complex in EAE lesions [123] (Figure 1B). This is significant since excitotoxic stimulation of AMPAR induces cell death in cultured neurons via nuclear translocation of GluA2-GAPDH complexes, where it likely promotes cell death via upregulation of the p53 pathway [124,125]. Interestingly, a cell penetrating peptide designed to disrupt the formation of GluA2-GAPDH complexes ameliorates both clinical disease and the degree of astrocyte reactivity in EAE [123]. In addition, the peptide reduces inflammation-associated changes in isolated cultures of astrocytes, including increased expression of GFAP and EAAT1/2 proteins, nuclear translocation of GluR2-GAPDH, and p53 activation [126]. These findings highlight AMPAR-mediated GluA2-GAPDH nuclear translocation and p53 activation as a potential mechanism connecting glial AMPAR to inflammatory excitotoxic disease states. Importantly, GluA2-GAPDH complexes are also enriched in MS lesions [123] suggesting that this complex may represent a promising therapeutic for inflammatory demyelination. In conclusion, while evidence linking astrocyte AMPAR activation to CNS disease is limited, subtle changes in the molecular characteristics of astrocytes following pathological AMPAR activation may contribute to the initiation and propagation of CNS injury and disease. In addition, AMPAR subunits may stimulate inflammatory disease processes through participation in novel protein complexes. Consequently, further work is required to investigate the links between CNS disease states, disturbances in the astrocyte AMPAR transcriptome, and the intracellular behaviour of GluA proteins.

## 3. Oligodendrocytes

Discovered by Pio Hortega in 1921, OL are the myelinating cells of the CNS [127]. Along development, three different waves of OL generation have been identified in rodent forebrain: the first one, arising from OL progenitors (OPC) stemming from the medial ganglionic eminence and anterior entopeduncular area, happens around E12.5, and is almost completely replaced by early postnatal stages [128]. This wave is followed by a second that originates in the lateral and caudal ganglionic eminences around day E16.5 [128]. Finally, around birth a new wave of OPC with a cortical origin populates cortical areas [128]; although some studies have pointed out that this cortical oligodendrogenesis may occur earlier in development [129]. Concerning cerebellar OL, their origin is mainly extracerebellar and with a likely source being OPC from the ventral rhombomere 1 (r1) that populate the cerebellum by day E18.5 [130]. Two different waves of OPC have also been described in the spinal cord [131,132]. The first one around E13, that produce most of the spinal cord OL, and the second, starting on E15.5, which contributes a smaller population of OL in spinal cord [131,132].

The maturation of the OL lineage is commonly divided into four stages: OPC, late OPC (also known as preoligodendrocytes, preOL), immature OL (iOL) and mature myelinating OL (mOL) [133,134,135]. OPCs are characterized by a bipolar morphology and the expression of markers like PDGF-Receptor α and NG2 [136,137]. These cells are able to migrate to different regions after damage or during development [138,139]. Of note, OPC remain abundant in the adult CNS where they retain the potential to differentiate into myelinating OL [15]. However, a substantial number of these cells remain undifferentiated, and it is this fact, coupled with their unique physiological connection to neural circuitry (see Section 3.2), that has led to the suggestion that they represent a distinct cell type, termed ‘NG2-glia’, with functions that extend beyond the generation of myelin forming cells [15,140,141]. Upon arrival at their target sites developmental OPC remain mitotically active, elaborate their processes, and lose their migrative properties before differentiating into preOL displaying immunoreactivity to the O4 monoclonal antibody [136,142]. A further stage of differentiation sees preOLs transition into iOL, which are post-mitotic cells with a highly complex morphological structure characterized by expression of galactocerebrosidase (Galc, recognized by the O1 monoclonal antibody) [142,143,144]. Finally, these cells wrap the axon, myelinating it as mOL [145,146].

Traditionally, the main function OL has been to myelinate the axon. Myelination is associated with an improvement of action potential transmission, increasing its speed and saving energy [147]. Thus, a lower velocity of conduction has been observed after demyelination [148], or in unmyelinated axons [149]. A recent hypothesis has proposed that myelination could contribute to a type of brain plasticity. Here, activity-dependent modulation of myelination on the most active fibers could, by altering their conduction velocities, provide a mechanism for coordinating the flow of information through neural circuits [10,150,151]. Myelin also protects axons from outside metabolites that could be harmful [152,153]. Besides isolating the axon, myelin also plays a role in the energetic metabolism of the axon as reviewed elsewhere [9]. These metabolic functions include involve the provision of energy substrates such as lactate or pyruvate, whose delivery to the axon contribute to the preservation of axon function and neuron survival [153].

### 3.1. Expression and Functional Properties of AMPAR in Oligodendrocytes

AMPAR are expressed at all stages of the OL lineage. OPC express all four GluA subunits, although GluA1 appears to be less prominent [154,155,156,157,158]. Analysis by patch-clamp recording from OPC in primary culture and acute brain slices indicate that OPC AMPAR exhibit rapid desensitisation kinetics, and a degree of Ca^2+^ permeability [155,156,159,160]. In support of this electrophysiological evidence, a number of Ca^2+^ imaging studies of OPC in cell culture and ex vivo preparations have revealed AMPAR mediated Ca^2+^ influx [140,155,161]. Given the prominence of GluA2 in OPC [155], and evidence showing the dominance of the Ca^2+^ impermeable Q/R edited form of GluA2 in hippocampal OPC [157] it has been proposed that OPC exhibit both Ca^2+^ permeable (GluA2 lacking) and impermeable (containing GluA2) AMPAR simultaneously [141]. A shift in potentiation to CTZ has been observed between postnatal day 5 and 12 suggesting a developmental increase in the proportion of AMPAR containing the Flip isoform [162]. Both Flip and Flop variants have been detected in the CG-4 OL cell line [163], but the relative expression of these variants in OPC in situ has not to our knowledge been reported.

Differentiated OL (iOL/mOL) continue to express GluA mRNA and protein, although the combination of subunits present appears to differ from that observed in OPC. RNA sequencing of OPC, iOL (also known as newly formed OL) and mOL indicates that all four GluA transcipts are down-regulated upon differentiation to the iOL stage, although the decline in GluA2 and GluA4 is less marked compared to GluA3 [164]. The decrease in expression continues with the transition to the mOL stage, although as discussed below it is notable that levels of GluA4 transcript remain relatively sustained [164]. In agreement with sustained GluA4 expression, AMPA receptor activation induces intracellular Ca^2+^ influx in mature OL isolated from the optic nerve and cortex [65,155]. At the protein level GluA2, 3 and 4 are detected in cultured iOL and mOL [155], and immunocytochemical staining has revealed expression of GluA4 in the soma and myelin of spinal cord OL [106], and in the soma and processes of CNPase-GFP expressing OL in optic nerve [165]. In the latter study, GluA2 was not detected in OL, but GluA3 protein expression in OL soma was inferred using anti-GluA2/3. In addition, a recent study in human white matter detected localisation of GluA4 protein to MBP^+^ myelin sheaths [166,167]. In contrast to these data, OL lineage cells within the forebrain white matter of immature rats exhibit immunoreactivity for GluA1, 2 and 3, and a brief expression of GluA4 protein on 04^+^ lateOPC between postnatal days 7-9, which was not observed on GalC^+^ iOL [168]. The expression and functional properties of OL lineage AMPAR are summarised in Table 2.

### 3.2. Oligodendrocyte AMPAR Functions Under Physiological Conditions

It is tempting to hypothesize that the pronounced alterations of AMPAR expression observed during OL differentiation [155,164] are related with the receptors role in OL maturation and myelination. Indeed, in line with evidence from other neural cell types, AMPAR have been implicated in developmental OL maturation and myelination [39,41,43,46,180] (Figure 1). AMPAR activation seems to have an inhibitory effect on OPC proliferation. This is observed by the reduction of the proliferative ratio after AMPAR stimulation in cell cultures [42], as well as its increase after AMPAR antagonism in ﻿organotypic cerebellar slice [41,43]. In agreement with this, a promotion of OPC proliferation has been studied after suppressing axonal release of glutamate [148]. By contrast, AMPAR activation appears to play a role in the recruitment of OPCs to target axons [148], perhaps via effects on OPC migration speed [37]. AMPAR also play a key role in OL differentiation, promoting the elongation and branching of OPC processes [41]. Indeed, this receptor seems to participate in the early stages of OPC lineage maturation rather than in developmental myelination [46,148], with alterations of this receptors inducing delays of myelination by reducing the number of mature oligodendrocytes [46].

The involvement of OPC AMPAR in developmental myelination has been challenged by recent in vivo work involving mice carrying a conditional deletion of OPC GluA2, 3, and 4 [46]. OPC AMPAR currents are completely abolished under these conditions, yet analysis of OPC in tissues from these mice failed to detect a change in OPC proliferation or differentiation [46]. Instead, the genetic ablation of OPC AMPAR resulted in a decrease in OPC survival. Importantly, parameters of myelination considered to be senstive to neuronal acitvity such as internodal length [181] and number [182] also remained unaltered. These findings are in contrast with data from larval zebrafish and rodent optic nerve where suppression of neuronal activity and attenuation of vesicular glutamate induce a measurable decrease in internode number and length respectively [182,183]. Technical differences in the approaches used to modulate glutamatergic actions on OPC could account for these contrasting results. Kougioumtzidou et al. [46] used a non-inducible Cre strategy to delete *Gria2* and *Gria4* in OPC on a constitute *Gria3*^−/−^ background. These tripple transgenic OPC lacked functional AMPAR through development, thus they would have been insensitive to all sources of glutamate-mediated AMPAR stimulation, be that vesicular or otherwise. One consideration is whether complete starvation of AMPAR-mediated signaling through the life-span may induce compensatory changes in OPC that mask the function of AMPAR in OPC maturation and myelination. In support of this view, recent work using OPC-specific retroviral-mediated modulations that alter OPC AMPAR only during postnatal development have identified an involvement of these receptors in OPC proliferation and differentiation, but not survival [180] (discussed in more detail below). With regard to the study by Kougioumtzidou et al. [46], it should also be noted that *Gria2, 3, and 4* lacking OPC would continue to receive glutamatergic stimulation from NMDAR [165,184,185], thus actvitity-dependent glutamate signaling could still influence OL maturation and myelination [185] (but see [186]). In contrast, Mensch et al. [183] and Etxeberria et al. [182] targeted glutamate release, rather than AMPAR expression, thus OPC in these studies could continute to receive stimulation from glutamate released by non-vesicular sources, which may act on both AMPAR and NMDAR. The use of an inducible-conditional *Gria 2, 3, 4* deletion, perhaps via a multiplex CRISPR-based knockout strategy, could help to bring further clarity to the role of AMPAR signaling in OPC maturation and myelination.

Notably, OPC AMPAR are activated by vesicular release of glutamate from unmyelinated axons in white and grey matter [141,187,188,189] (Figure 1A and Figure 2A). The function of these neuro-glial synapses is unknown, but it is hypothesised that they may signal levels of activity within neural circuits, perhaps allowing OPC to regulate their proliferation or differentiation at sites of increased activity [141,190]. In agreement with this idea, AMPAR-mediated input declines upon differentiation of OPC [191], and synaptic activity can induce Ca^2+^ influx into OPC via AMPAR [159,160], thus the synaptic activation of pro-differentiation Ca^2+^-dependent intracellular signals seems a possibility. However, recent evidence suggests a role for axon-OPC synapses in regulating proliferation but not differentiation [180]. In this work increases in the Ca^2+^ permeability of OPC AMPR via OPC specific expression of either non Q/R edited GluA2 subunits, or a “pore dead” GluA2 construct, promoted OPC proliferation without affecting differentiation or survival. Thus neuronal activity may influence OPC proliferation via the activation of OPC AMPAR and the subsequent activation of Ca^2+^-dependent signaling pathways. Interestingly, an additional strategy that reduced the proportion of Ca^2+^ permeable AMPAR in OPC without affecting GluA2 channel properties caused an increase in the size of the OPC population without altering proliferation or survival [180] suggesting further complexities in the influence of AMPAR on OPC development. Contrasts between these findings, and those indicating an enhancement of OPC proliferation following AMPAR antagonism in cerebellar slice cultures [41,43] may be explained if bath applied AMPAR blockers, as used on ex vivo slices, affect additional mechanisms that impinge on OPC functions. One possibility, as highlighted previously [41], would be an effect on neuronal synapses whose inhibition would be expected to produce similar effects to that seen when neuronal activity is blocked pharmacologically. Of note, both TTX and the AMPAR antagonist GYKI induce a similar stimulation of OPC proliferation in cerebellar slice cultures [41]. Taken together there is considerable evidence that OPC AMPAR, including those recruited via neuron-OPC synapses, exert influences on OPC migration, proliferation and survival during CNS development (Figure 1A).

Interestingly, a large numbers of OPC, or NG2-glia, persist in the adult CNS where they continue to receive synaptic input from neuronal circuits [reviewed by 182]. These NG2^+^ cells seem able to respond to this activity since, like their developmental counterparts [161], they exhibit activity-dependent and neurotransmitter receptor dependent Ca^2+^ transients [192]. These observations, and morphological data showing that their processes make intimate contact with multiple neuronal and astrocyte elements, are suggestive of specialized functions within the CNS [192]. Indeed, it has been proposed that NG2^+^ cells might regulate glutamatergic synapses by modulating postsynaptic AMPA [193], although this idea remains controversial at this time [194]. Aside from a role in remyelination (Section 3.2) other functions for OPC/NG2-glia in the adult CNS remains an open question.

Regarding differentiated OL, both iOL and mOL continue to express AMPAR (Section 3.1), and GluA4 has been detected in rodent spinal cord myelin [106] and human cortical white matter [167]. Ca^2+^-permeable AMPAR containing GluA4 may contribute to activity-dependent signaling between axons and myelin. Electrical stimulation of an ex vivo optic nerve preparation induces Ca^2+^ signaling in myelin that is blocked by NMDAR and AMPAR antagonists [109]. These data suggest that AMPAR activation may provide a depolarizing stimuli that relieves Mg^2+^ blockade from NMDAR, and indeed imaging in zero Mg^2+^ revealed a subtle NDMAR-dependent elevation in Ca^2+^ [109]. This form of activity-dependent signaling involves vesicular release of glutamate: Ca^2+^ signals were blocked by bafilomycin and tetanus toxin, both of which disrupt vesicular release; and enhanced by hypertonic sucrose stimulation, which encourages vesicle release. Whether or not this form of axon-myelin signaling occurs in other regions of the CNS, and what function it may play in regulating axon-OL interactions remains unclear. However, it has been proposed that an ‘axo-myelinic’ synapse may enable mOL to sense activity in target axons, allowing them to adjust the provision of metabolic support [195], perhaps via the provision of lactate [196], or to fine tune myelin internode parameters such as length and thickness in response to changes in demand [195].

### 3.3. Oligodendrocyte AMPAR in Pathology

The prominent expression of Ca^2+^ permeable AMPAR in the OL lineage places these cells at threat of injury from excitotoxic conditions (Figure 2B). Indeed, AMPAR-mediated excitotoxicity has been described in numerous in vitro studies using cultures of both OPC/preOL [45,170,171] and differentiated OL [65,163,197]. Importantly, excitotoxicity in these cells is associated with excessive Ca^2+^ influx [65,171], thus expression of Ca^2+^ permeable AMPAR, as described in Section 2.2, appears to render these cells vulnerable to high levels of extracellular glutamate. In support of this idea, specific antagonists of Ca^2+^ permeable AMPAR reduce injury in OL subjected to oxygen glucose deprivation [171], while forced expression of Q/R edited GluA2 subunits protected OPC from an AMPAR-induced excitotoxic injury [173]. Given the links between Ca^2+^ influx and cellular injury responses in OPC, the degree of Ca^2+^ permeable AMPAR expression in OPC would be expected to regulate vulnerability to excitotoxic injury. In this respect, it is interesting that group 1 mGluR activation leads to an increased surface expression of Ca^2+^ permeable AMPAR in OPC [198]. This mechanism, which involves specific TARP-dependent trafficking of the Ca^2+^ permeable GluA4 subunit, may act to amplify the sensitivity of OPC to elevated levels of extracellular glutamate. However, at this time the links between mGluR activation and OPC excitotoxicity remain unclear.

AMPAR-mediated injury has been observed in a number of in vivo models involving OL and myelin injury. For example, the AMPA antagonists NBQX reduces OL and myelin loss in rats subjected to a weight-drop spinal cord contusion injury [179], and as discussed below, ameliorates inflammatory demyelination in the EAE model of MS [119,120]. These observations are significant since excitotoxicity is implicated in a number of pathologies (described in Section 1.3) including Cerebral White Matter Injury (WMI) and MS [199,200]. In premature infants WMI includes different neuropathological cerebral white matter [201], the survivors of which eventually develop long-term neurological disabilities [199,202]. Within the framework of pre-term conditions, H-I damage has been specifically correlated to oligodendrocyte maturation, with preOL being identified as being particularly vulnerable in several species [115,203,204,205] including human [206]. For a detailed discussion of perinatal WMI see the recent review by Back et al. [199]. Importantly, within the OL lineage, preOL in developing white matter exhibit the greatest abundance of GluA4 [168,207], a Ca^2+^ permeable subunit highly expressed in neural cells exhibiting vulnerability to excitotoxic death [208]. Importantly, topiramate and NBQX reduce OL death in rodent models of neonatal H-I injury [172,178]. Thus, the protection of OL via the targeting of Ca^2+^ permeable AMPAR, or relevant downstream pathways, represents a potential strategy for the prevention of WMI in pre-term infants. However, despite these promising pre-clnical findings, it should be noted that other pathogenic events are likely to be relevant since preOLs are also very sensitive to increased levels of TNFα and oxidative stress, both of which are characteristic of WMI [209,210,211].

Related results are obtained after stroke in neonatal and adult rodent models. In both cases, cerebral ischemia is followed by a decrease in the mOL population [212,213] as well as OPC proliferation and migration into the damage areas [213,214]. mOL death is reproduced in vitro by AMPA but not NMDA administration, and is reduced by AMPAR antagonist after oxygen and glucose deprivation [197,215]. The administration of AMPAR competitive antagonist, SPD 502, mimics this oligoprotection after cerebral stroke induced in adult rats [216]. Interestingly, this effect is regionally restricted since the oligoprotection is specific to cortical OL. As mentioned above, brain OL are heterogenous both in respect of their developmental origins, and their differentiation rates in grey and white matter [217,218]. Therefore, deeper studies to analyze how AMPAR modulation affects specific OL populations are warranted if AMPAR-targeting therapeutics are to be developed further.

A number of observations link glutamate dysregulation and OL AMPAR to pathology in MS. First, MS is characterized by high levels of glutamate within CSF [5,62]. Second, an increase in levels of the Ca^2+^ permeable subunit GluA1, but not GluA2, in oligodendrocytes located next to MS plaques has been observed in post-mortem CNS samples of MS patients [219]. Third, a greater level of GluR2–GAPDH complexes are detected in MS plaques [123], and disruption of the GluA2-GAPDH complex has been shown to produce a therapeutic/protective action in EAE [123] (see Section 2.3 for a related discussion on this topic). Fourth, Ca^2+^ permeable AMPAR are implicated in CNS pathology in EAE where OL death, axonal damage and demyelination are reduced in mice lacking Gria3, the gene encoding the Ca^2+^ permeable GluA3 subunit [220]. In agreement with these latter findings, other work in the EAE model encourages the use of AMPAR as therapeutic targets in MS since blockade of these receptors via subcutaneous injection of NBQX reduces clinical disease and protects OL [119,120]. These promising preclinical results from models of MS [221], and also stroke [216,222], must be weighed against the negative findings from clinical studies that have observed worsened outcomes following the use of AMPAR-targeting drugs in patients with acute ischemic stroke, [223,224]. These disappointing results may be explained by the low specificity of the drug used, ZK200775, and the wide expression of these receptors in CNS circuitry, where their inhibition may be expected to produce numerous unwanted side effects that may interfere with any potential therapeutic benefits. As discussed above (Section 3.2) AMPAR influence the development and survival of OL. Consequently, protection of OL from excitotoxic conditions associated with CNS inflammation may not be achieved without compromising the regenerative capacity of OPC (but see [172]). Nonetheless, glutamate dysregulation plays wider roles in MS pathogenesis via actions on T cell function such as migration and cytokine secretion, and even glutamate release [5], thus therapies focused on controlling glutamate levels may provide significant benefits that extend beyond oligodendrocyte protection.

The activation of OPC AMPAR by axonal synaptic input may play a role in regulating OPC-mediated remyelination (Figure 1). New OPC recruited to lesions express AMPAR, which may be involved in both migration and the early stages of myelination [39,148]. Within the caudal cerebellar peduncle, axons demyelinated by infusion of ethidium bromide establish de novo glutamatergic synapses with recruited OPCs, while infusion of antagonists of specific voltage-gated Ca^2+^ channels, selected to specifically act on axonal channels, reduce the degree of remyelination [148,176]. Thus, synaptic activation of OPC AMPAR appears to be important for remyelination. Another study tracked the occurrence of synaptic inputs on OPC following lysolecithin lesions in the corpus callosum [176]. Here the authors report a disruption of synaptic input shortly after demyelination that correlates with a reduction in immunohistologically identified axonal synaptic connections on proliferating OPC in the lesion. Interestingly, a recovery in synaptic innervation coincides with a reduction in OPC proliferation, suggesting that AMPAR activation may inhibit cell division [176]. This observation agrees with recent studies indicating a role for synaptic Ca^2+^ permeable AMPAR in stimulating OPC proliferation [180], and with data from brain slice cultures and in vivo studies that show that blockade of axonal activity increases OPC proliferation [41,148,225,226]. However, blockade of activity in ex vivo brain slices also produces an increae in OL differentiation [41] that was not observed under in vivo remyelinating conditions [148]. These contrasting outcomes may be explained by differences in experimental models (ex vivo vs. in vivo) and the developmental status of the tissues examined (developing white vs adult remyelinating). Despite these differences the data from these studies, and many others from a variety of different CNS systems, clearly indicate an important role for axonal activity and AMPAR in the regulation of OL myelination [113,181,182,183,225,227] (Figure 1).

In conclusion, the excessive activation of OL AMPAR poses numerous threats to the viability of OL and the myelin they support. This loss of myelin compromises neurological function in a wide range of CNS disease states, thus therapies that protect OL from excitotoxic AMPAR-mediated injury are an important clinical goal. Nevertheless, AMPAR are powerful regulators of OL development and survival, and may also play unidentified functions in adult-NG2-glia. Therefore, AMPAR-targeting therapies capable of protection OL may induce unwanted side effects, and potentially hinder other aspects of OL regeneration and myelin repair. Further basic and pre-clinical research will be necessary to fully identify the functions of OL AMPAR, and determine the benefits and feasibility of targeting these receptors in a therapeutic context.

## 4. Microglia

Microglia are highly abundant CNS glia, whose origin within the embryonic yolk sac [6], and macrophage-like functions, distinguish them from all other parenchymal glia in the nervous system. In the healthy brain microglia exhibit highly motile processes that scan the local tissue landscape, seemingly searching for signs of disease or injury whose detection may trigger them into acquiring an activated amoeboid phenotype capable of launching inflammatory responses and engaging in phagocytic activity [228]. Microglia are considered to serve a number of protective roles that collectively help to maintain a healthy environment with the CNS. These functions include the removal of dead and dying cells, the regulation of synapse number via pruning of excess connections, and the production of various neurotrophic factors capable of influencing the development and survival of other neural cells [22]. In addition, microglia express major histocompatibility complex (MHC) I and II molecules, particularly under pathological conditions including perinatal hypoxia, thus a role in the detection of pathological conditions and the stimulation of adaptive immune responses are ascribed to these cells [229]. In addition to protecting the healthy CNS, microglia contribute to the generation of pathological conditions via the synthesis and release of cytotoxic molecules such as nitric oxide, reactive oxygen species and proinflammatory cytokines [230]. They also contribute to CNS repair processes by releasing neuroprotective factors, reducing inflammation, and encouraging regenerative processes such as OPC differentiation and remyelination [230]. These contrasting roles in CNS protection and injury have frequently been cast within a model where microglia exhibit polarised states, the so-called M1 (pro-inflammatory)/M2 (anti-inflammatory pro-repair) phenotypes [231]. However, a number of observations, such as the co-existence of cardinal M1/M2 molecules within microglia in vivo, have lead this dichotomy to be described as unsuitable and unhelpful for the understanding of microglial biology within the intact CNS [232]. Nevertheless, microglia certainly exert profound and varied influences in the context of CNS protection, immune activation, injury and repair, whose regulation by AMPAR may contribute to the induction and progression of a number of disease states.

### 4.1. Expression and Functional Properties of AMPAR in Microglia

Consistent evidence for function AMPAR expression in microglia has been reported in experiments on cultures of cortical rat microglia [233]. Reverse transcription PCR indicates that these cells express GluA transcripts 2, 3 and 4 [234], although a subsequent study using more sensitive quantitative RT-PCR method detected GluA, 1, 2 and 3 mRNA [235]. Expression of GluA1 has also been shown at the protein level in rat cortical microglia via immunocytochemistry [236]. Functionality for these AMPAR has been confirmed by patch-clamp analysis where CNQX sensitive glutamate currents are detected [233]. These currents were potentiated by CTZ, and showed little Ca^2+^ permeability [233], consistent with the presence of GluA2 transcripts. Interestingly, experiments with AMPAR modulators show that microglial glutamate currents exhibit a greater degree of potentiation to CTZ compared to PEPA [234]. CTZ is selective for Flip variants, while PEPA exhibits a greater affinity for Flop subunits, thus these data suggest a dominance of Flip splice variants in microglia [234]. In line with this finding transcript analysis in the same study revealed a predominance of GluA1-3 Flip variants, a configuration that may render cells more sensitive to excessive glutamate levels (Section 1.2). In contrast to the in vitro studies described above, evidence for microglial AMPAR expression in vivo is more uncertain. AMPAR expression has been detected by immunostaining for GluA2/3 and 4 in OX-42 cells in rat forebrain sections [237], although this work was done under permeabalising conditions hence it is not clear if the immunosignals represent surface expressed receptors. Importantly, immunostaining did not identify receptor subunits on the surface of retinal microglia, and patch-clamp recordings from microglia have failed to detect glutamate-mediated currents in a range of CNS tissues including spinal cord, hippocampus and retina (reviewed in [238]). Interestingly, hypoxia causes a marked increase in microglial GluA2-4 protein expression within forebrain periventricular white matter [237], and hippocampal microglia express GluA4 protein only after exposure to an ischemic injury [239]. These in situ observations suggest that microglial AMPAR expression may be low or absent under healthy conditions, but may be induced in microglia activated under pathological conditions [238] (Figure 2). Furthermore, the contrast between these findings, and those from in vitro studies, suggest that the physiological properties of microglia may differ significantly under cell culture conditions. Indeed, differences in the expression of voltage-gated K^+^ channels are observed when patch-clamp recordings are made from microglia in actute vs cultured hippocampal brain slices [240]. A summary of findings on the expression and functional properties of microglial AMPAR are summarised in Table 3.

### 4.2. Microglial AMPAR in Pathology

Evidence linking AMPAR activation to microglial functions under physiological conditions appear to be limited to effects on chemotaxis, where glutamate stimulates a directed-migration of microglia in cell cultures and spinal cord slices via AMPAR receptors [117]. In contrast, several interesting actions are associated with the activation of microglial AMPAR under pathological conditions that are linked with excessive glutamate signaling. Studies using a rat model of periventricular white matter (PWM) injury, and microglial cell cultures exposed to hypoxia, have identified links between hypoxia-induced elevations in glutamate, and the regulation of protective and inflammatory microglial-derived substances [237] (Figure 2B). This work shows that hypoxia causes increases in PWM concentrations for both glutamate and IGF-1, a neurotrophic factor associated with the protection of neurons [241] and oligodendrocytes [169,170] following H-I injury. Importantly, elevated IGF-1 protein is localised to microglial with an activated amoeboid morphology that also exhibit enhanced expression of GluA2, 3 and 4 subunits [237] (Figure 1B). While increased expression of IGF-1 may play a protective role following this injury, the increase in AMPAR expression in these cells appears to prime them for a more pathological response since a subsequent exposure to glutamate simultaneously attenuates IGF-1 production, while promoting TNF-α release and IL1-beta [237]. This inflammatory response is mechanistically linked to the reduced levels of IGF-1 seen after glutamate stimulation since knock-down of IGF-1 gene expression promotes TNF- α and IL-1 beta gene expression. Overall, the study by Sivakumar et al. [237] suggests that increased expression of microglial AMPAR in hypoxic PWM tissue sensitises microglia to glutamate leading to a regulatory response that reduces protective IGF-1 release while increasing the release of inflammatory mediators.

Other evidence suggests that microglial AMPAR activation, and a subsequent production and release of TNF-α, may contribute to the propagation of CNS disease states (Figure 2B). First, cell culture and in vivo experiments show that the activation of microglia AMPAR stimulates the production and release of TNF-α [234,237]. Second, TNF-α signaling is heavily associated with a number of glial-dependent actions, including the emission of glutamate via gap-junction hemichannels from microglia [242] and astrocytes, Fas ligand release from microglia, and the downregulation of EAAT2/GLT1 glutamate transporters in astrocytes, which trigger neurotoxicity in the CNS [2]. Third, TNF-α release from astrocytes stimulates the trafficking of AMPAR to the surface of hippocampal and cortical neurons, rendering them more sensitive to excitotoxic conditions involving excessive levels of extra cellular glutamate [243]. Fourth, conditioned medium containing TNF-α released from kainate-stimulated microglia induces the upregulation of voltage-gated Ca^2+^ channels and NMDA-type glutamate receptors in hippocampal neurons, and is associated with neuronal apoptosis [244]. In summary, microglial AMPAR-mediated TNF-α release, acting through a range of autocrine and paracrine routes, are positioned to elevate extracellular glutamate concentrations, release apoptosis inducing signals, and increase the sensitivity of neurons to excitotoxicity. As outlined in Section 1.3, excitotoxic injury has a link to a wide range of CNS injury and disease states [1,4], thus further investigation of the influence of the microglial AMPAR-TNF-α axis, and its actions in propagating excitotoxic conditions, are warranted. In this regard, a promising area for research can be found in the context of MS, where impaired cognitive function may be linked to pathological alterations in synaptic structures that arise early in the course of the disease [245]. Research using the EAE model of inflammatory demyelination shows that TNF-α contribute to the mechanisms driving these synaptic dysfunctions, at least within the striatum, where these pathophysiological actions are correlated with the activation of microglia and astrocytes [246,247]. While the involvement of microglial AMPAR is in these actions is unknown, the established links between their activation and the release of TNF-α [234,237] indicates that they could be capable of amplifying an initial inflammation-induced elevation in extracellular glutamate into additional excitotoxic insults.

Microglial AMPAR have also been implicated in neurodegenerative disease processes through a series of cell culture studies examining the regulation of GluA subunit surface expression in activated microglia [248]. Here, the authors examined the hypothesis that down-regulation of GluA2, as is observed in a number of neurodegenerative conditions including MS [249], drives neurotoxicity via increased release of pro-inflammatory cytokines from microglia. Analysis of cultured microglia generated from a global GluA2 null line exhibited a greater degree of Ca^2+^ permeability, and enhanced TNF-α production following AMPAR activation. In line with this data, conditioned medium from GluA2 ^-/-^ microglia exerted a greater neurotoxic effect on cultured neurons. Thus, as proposed by Noda and Beppu [250], the loss of microglial GluA2 under pathological conditions may serve to accelerate neuronal injury and loss via alterations in the subunit composition of their AMPAR that favour Ca^2+^ entry and inflammatory cytokine release. While this hypothesis provides an attractive link between microglial AMPAR function and disease, further work should be performed using a conditional deletion of GluA2 in microglia to refine the loci of action and confirm the relevance of this mechanism in relevant in vivo disease models.

In summary, microglial are equipped with AMPAR whose activation may help to synchronise the production and release of inflammatory molecules to inflammatory excitotoxic conditions within the tissue. These actions could trigger de novo excitotoxicity or may contribute to the propagation of an ongoing disease state. Based on these actions, therapies targeting microglial AMPAR represent a valuable ambition, the achievement of which may provide a means to modulate neurotoxicity in a number of neurodegenerative CNS conditions.

## 5. AMPAR in Other Glial Cells

AMPAR are expressed in glial cells beyond the principle types described in Section 2, Section 3 and Section 4, yet their functions in these ‘other glia’ are less characterized. Nonetheless, these glia, which in this review include Radial Glia, Schwann Cells, Satellite Glia and Enteric Glia, have important functions in both CNS and PNS. Therefore, AMPAR might yet play significant roles in their behavior under physiological and pathophysiological conditions. Here we present a brief overview of AMPAR in these other glial cells. See Table 4 for a summary of the expression and functional properties of AMPAR in these glial cells.

### 5.1. Radial Glia

Radial glia (RG) gives rise to astrocytes, neurons, and oligodendrocytes during embryonic development [16,262,263]. In primary cell cultures of RG-like neural progenitor cells (NPC) qPCR reveals a high level of GluA1 mRNA, and lower levels of GluA2, 3 and 4, while immunostaining detects GluA1, 2 and 3 protein expression on RG/NPC expressing the glial marker GLAST [251]. The functionality of these AMPAR is evident in Ca^2+^ imaging data where transient AMPA induced elevations in intracellular Ca^2+^ are enhacned by CTZ and reduced by philantotoxin, a specific antagonist of Ca^2+^ permeable AMPAR [251]. In contrast, another study revealed high levels of GluA3 and 4 mRNA, and an absence of GluA1 and 2, in NPC [252]. In agrement with this data, immunohistochemical staining reveals strong GluA3 and 4 localisation to RG in the developing white matter of rodents [168], while GluA4 is localised to RG in fetal human RG [207]. Differentiation of RG/NPC into astrocytes or neurons involves an increase of GluA2 and a decrease of GluA3/4, thus suggesting an increase in the proportion of Ca^2+^ impermeable AMPAR [168,207,252,253,264]. These findings suggests a role for glutamate acting via AMPAR in RG proliferation and differentiation. In agreement with this idea, AMPAR are involved in the regulation of RG processes length and RG motility [265].

RG-like cells are also present in the adult subgranular zone of the hippocampus [166,253]. In contrast to developmental RG, these adult RG-like cells possess AMPAR that are mainly composed of GluA2 subunits, and which are expressed in the cell’s processes but not in the soma [166]. The administration of kainate promotes RG-like cell proliferation and survival, both in vitro, and in vivo, in an animal model of status epilepticus [253].

Very little is known about AMPAR expression in ependymal cells derived from radial glia, although GluA2/3 expression has been found in cell bodies and proximal thick processes of tanycytes [254].

### 5.2. Schwann Cells

As mentioned in the introduction (Section 1.1), Schwann cells are the myelinating cells of the PNS. As in the CNS, PNS axons extend significant distances, thus in addition to the benefits associated with OL myelination, such as enhanced conduction velocity and axonal isolation (Section 3), trophic support arising from myelinating Schwann cells is likely to be important for sustaining the viability of axons located long distances from the cell body [152]. Distinct from the myelinating forms, the perisynaptic Schwann cells (PSCs) are non-myelinating cells that are part of the neuromuscular junction (NMJ) formed between the presynaptic nerve terminal and the postsynaptic specialization [264]. These cells plays an important role in the growth and maintenance of NMJs as well as in the modulation of its synaptic properties [264,266,267]. In comparison to OL, the very little less is known regarding the expression and function of AMPAR in SCs.

During development mRNA expression of all AMPAR subunits is detected in mouse sciatic nerve, although it is not clear if this expression arises from the axon or glial cells [256,268]. While GluA2/3 and GluA4 are detected by electron microscopy in Schwann cells of the vestibular system of rats and guinea pigs; only GluA1 and GluA4, but no GluA2 or GluA3, protein expression are found in primary SC culture [255,258]. Patch-clamp recording from developing SC in peripheral nerve tissue and cell cultures exhibit functional Ca^2+^ permeable AMPAR [256,257]. A detailed study performed in sciatic nerve preparation of mouse pups at embryonic day 16th–18th or postnatal day 0–2nd has proved that AMPAR are modulated along development, with functional AMPAR only expressed in developing SCs while more mature myelinating SCs are unresponsiveness to glutamate [256]. Furthermore, the amount of AMPAR might also be modulated in developing SCs [256].

However, the AMPAR role in SCs is still not clear. Metabotropic glutamate receptors activation induces SCs proliferation, whereas migration induced by glutamate are also NMDAR-dependent [268,269,270]. AMPAR activation on PNS myelinated axons induced an increase of axoplasmic Ca^2+^, although myelin abnormalities are only observed after prolonged activation of NMDA, but not AMPAR [271]. AMPAR activation has also be found to control ATP release after glutamate stimulation in SCs culture [258].

### 5.3. Satellite Glia

Among satellite Glia cells (SGCs), those of the sensory ganglia has been the most studied. [25]. SGCs are in close contact with neurons, ensheathing them and playing a role in neurotransmission and glutamate homeostasis [25,259,260,272]. To that end, SGCs express several glutamate transporters, metabotropic glutamate receptors, NMDAR, KR and AMPAR [259,260]. Regarding AMPAR, SGCs express mainly GluA4 and in lesser quantities GluA2/3 but no GluA1 [259,273]. AMPAR activation induces a rapid Ca^2+^ influx in SGCs inline with their expression of GluA4 [259]. In addition, a role for SGC NMDAR has been described in pathological situations like hyperalgesia [274]. It is therefore tempting to hypothesize that AMPAR could also play a role in SGCs response after damage.

### 5.4. Enteric Glia

Enteric glia are non-myelinating glial cells of the enteric nervous system, localized within the wall of intestines they are critical in the control of gut motility [275,276]. Despite showing some similarities to astrocytes, enteric glia display a unique transcription profile [275,276]. The connection between enteric neurons and enteric glia is not well characterized, although ionotropic glutamate receptors have been found in both cell types [261,275]. Concerning AMPAR only GluA1 and GluA3 subunits have been found in enteric glia, but the Ca^2+^ permeability of the functional AMPAR has not been fully elucidated yet [261]. AMPAR are involved in excitotoxic damage to enteric neurons in studies performed in myenteric ganglia [277,278]. Although it is unclear whether these pathological actions involve AMPAR located on enteric glia, their expression of GluA proteins suggests they may also be targets during ischemic injury in these enteric tissues.

## 6. Cannabinoids and AMPA Receptor

Several studies have pointed out that cannabinoids and the Endocannabinoid System (ECS) might modulate glutamatergic transmission, including AMPAR. This modulation has been principally studied in neurons, although glial receptor involvement has also been described [279,280,281].

The medical use of Cannabis sativa has been explored for millennia worldwide [282]. Modern research in the cannabinoids field started during the last century with the isolation of Δ^9^- tetrahydrocannabinol or THC [283], a discovery that eventually lead to the recognition of the ECS. The ECS is an endogenous system that plays important physiological roles, modulating neuronal synapses, the immune response or energy and metabolism regulation, among others [284,285,286,287,288].

As has been recently reviewed, the ECS modulates OL lineage cells and myelination [289]. Different elements of the ECS are expressed in all OL stages, and observations that the ECS is modulated during OL maturation, suggest that they may play a role in OL differentiation [290,291]. The endocannabinoid 2-arachidonoylglycerol (2-AG) and cannabinoid receptors CB_1_ and CB_2_ activation promote OPC proliferation, migration and maturation, and oligodendrocyte myelination [290,291,292,293,294,295]. Indeed, different pharmacological studies have proved that the reduction of 2-AG levels, or blocking either CB_1_ or CB_2_ reduces myelination, both in vitro and in vivo [291,296].

Based on the links between the ECS and OL lineage functions described above, the modulation of the ECS has been studied as a possible treatment for demyelinating diseases like MS [289]. A cannabinoid derived drug, Sativex, is approved to treat MS spasticity and pain [297,298], and in different animal models of MS, cannabinoid treatment based on cannabidiol solely, or cannabidiol plus THC (Sativex), reduces the myelin injury, inflammation and functional impairment [299,300,301]. The modulation of 2-AG levels effectively reduces motor symptoms, along with inflammation and demyelination [302,303]. Some of this protective effect is mediated by cytosolic Ca^2+^ modulation. Either the inhibition of 2-AG degrading enzyme, or the direct administration of 2-AG preventing intracellular Ca^2+^ increase via AMPARs, and the subsequent pattern of mitochondrial dysfunction and ROS increase [302]. Similar results are obtained in cultured OL when CB_1_ receptor agonist are administered after AMPAR activation, or K^+^- induced depolarization, with both producing a reduction in intracellular Ca^2+^ [302,304] (Figure 1B). These processes might involve the activation of Gi/0 proteins, the blockade of Kir or voltage-gated Ca^2+^ channels (VGCCs) [304,305]. The administration of the phytocannabinoid cannabidiol (CBD), a promising therapeutic molecule, also modulates intracellular Ca^2+^ via mechanisms involving mitochondria, and can, under specific conditions, induce OL cell death [306]. In spite of the controversy concerning the deleterious effect of CBD on OL cell cultures, some authors have identified a dose-dependent response [306,307]. Furthermore, CBD has been reported to be oligoprotective after an inflammatory damage [307], and to reduce excitotoxicity in several pathologies such as stroke or neonatal hypoxia-ischemia [58,308,309,310]. Results of our group have proved this dual effect. While the exposure of mouse organotypic cerebellar slices to CBD resulted in a reduction of the OL population; CBD applied before and during an excitotoxic insult was able to reduce OL cell death (Ceprian M, Ng J, Fulton D, unpublished). Promising oligoprotective findings from CBD treatments suggest that more research in this area are warranted. An important area for this work relates to the mechanism of CBD action on glia, which at this time remains unclear.

After an excitotoxic insult, there is an increase of endocannabinoids and endocannabinoids-like molecules, probably released by neurons [308,311]. Interestingly, the administration of the endocannabinoids 2-AG or anandamide (AEA) or synthetic cannabinoid agonist, HU-210, reduces cell death after AMPA administration in a mixed culture of astrocytes and neurons [221,311]. HU-210 neuroprotective mechanism are also seen in vivo and requires astrocytes and CB_1_ and CB_2_ activation [221]. AEA also exerts its neuroprotection via CB_1_ and CB_2_ receptors, and by preventing the AMPAR-induced downregulation of astrocyte glutamate transporters GLAST and GLT-1, as evidenced in both primary cultures of astrocytes, and an in vivo model of MS [311] (Figure 1B). In agreement with these results, the agonism of both cannabinoid receptors by WIN55,212-2 increases GLAST and GLT-1 expression in the spinal cord of EAE induced animals [312], while another study in the EAE model has found a decrease of AEA and 2-AG, which due to the actions on GLAST and GLT-1 described above, would be expected contribute to glutamate excitotoxicity [313] (Figure 1B).

Finally, the administration of the phytocannabinoids THC and CBD modulate the expression of neuronal GluA2-AMPAR in rodent models of drug abuse [314,315]. Further research to analyze if a similar AMPAR subunit modulation occurs after cannabinoid administration in excitotoxic models could help to elucidate cannabinoid’s protective action on glial cells.

## 7. AMPAR-Stimulated Gene Expression in Glial Cells: Contributions to Injury and Disease?

Activity-dependent receptor-mediated gene expression, particularly immediate early genes (IEGs), regulates key functions in the CNS including development and synaptic plasticity [316,317]. Gene expression in glial cells can also regulated by neuronal activity, for example in astrocytes where neuronally derived notch signals regulates a broad range of genes leading to alterations in astrocyte development, metabolism and neurotransmitter uptake functions [318]. As described in Section 2.1 astrocytes express functional AMPAR that allow them to respond to glutamate released from neuronal synapses. Given the broad range of astrocyte genes regulated by neuronal activity [318] it is tempting to speculate on the potential of AMPAR to contribute to these actions. For example, AMPAR activation on Bergmann glial cells leads to a downregulation in transcription of the glutamate transporter GLAST via a mechanism involving Ca^2+^ influx, PKC signaling and the activation of the IEG *c-jun* [99]. GLAST downregulation was observed with prolonged exposures to glutamate. Triggering of this regulatory response during periods of elevated extracellular glutamate, as occurs in pathological conditions such as MS [121], stroke and hypoxic-ischemia [4], could therefore lead to an amplification of the pathophysiological levels of glutamate (Figure 2).

AMPAR have also been shown to regulate IEG in cortical cultures of OPC [44] where stimulation of AMPAR induced a Ca^2+^-dependent upregulation in *ngfi-a* and other IEGs (Figure 1A). *Ngfi-a* (also known as *zif-268*, *egr-1 and krox-20*) is implicated in regulation of the cell cycle [319], and is induced dramatically in the brain in a depolarisation-dependent manner [320]. Thus *Ngfi-a* activation by AMPAR may contribute to the regulation of OPC proliferation observed following modulation of neuronal activity and AMPAR [41,148,210]. AMPAR stimulation inhibits OPC proliferation [210], thus pathological elevations in glutamate concentrations could act to reduce the supply of remyelinating OPC. As discussed in Section 3.3, excessive AMPAR activation injures OL lineage cells via Ca^2+^-dependent mechanisms involving mitochondrial stress and the induction of pro-apoptotic Bcl-2 molecules [65,169,321]. Apoptosis is closely regulated by the induction of pro- and anti-apoptotic Bcl-2 genes [322,323], so the pathways connecting pathophysiological AMPAR stimulation may provide an interesting range of targets for therapeutic research. Related to this idea, we recently examined the transcriptional events induced in OPC following pathophysiological AMPAR stimulation [45]. To focus our work we searched for potential regulators of *Gria4,* the gene encoding GluA4, via an in silico analysis. *Gria4* was an attractive target since it has prominent expression in OPC, and has been linked to excitotoxic injury in other cell types [208]. From among the set of candidate regulators we selected NF-Y subunit b (NF-Yb), a member of the NF-Y complex whose activity is closely associated with the regulation of apoptototic cell death [88,324]. Experiments using an OPC cell line revealed that excitotoxic AMPAR stimulation altered NF-Yb expression, modulated its binding to regulatory regions within *Gria4*, and altered the expression of *Gria4* transcripts. Thus NF-Yb is regulated by pathophysiological stimulation of AMPAR leading to alterations in the expression of target genes. Further experiments using primary OPC showed that excitotoxic stimulation produced a parallel increase in the expression of NF-Yb and its target gene *Gria4*. Interestingly GluA4 protein is upregulated in OL in an in vivo hypoxia model [237], thus it is tempting to speculate on the involvement of NF-Yb in these actions. NF-Yb is linked to the control of apoptosis [88,324] thus we performed a transcriptomic analysis to identify genes that were differentially regulated by NF-Yb modulation and excitotoxicity. For this analysis we compared the effects of a treatment with Garcinol, a compound that both blocked NF-Yb binding to *Gria4* and reduced cellular viability, with an excitotoxic AMPAR stimulation proven to injure OPC. Both treatments induced transcriptional regulation in the same set of apoptotic genes underscoring the link between NF-Y function, excitotoxic injury, and apoptosis in OPC (Figure 1B). As described in this review, AMPAR are ubiquitous in neurons and glial cells so the clinical use of systemic AMPAR blockade is likely to produce numerous side effects that may complicate the evaluation of therapeutic outcomes. Cell-specific targeting could reduce these problems, but the technologies available are not translatable, and in any case, AMPAR influence numerous physiological functions in glial cells whose modulation may worsen disease conditions. Consequently, molecular targets downstream of pathological AMPAR activation, for example those contained within the NF-Y transcriptome, represent an attractive proposition for research aiming to provide protection against pathological conditions involving AMPAR-mediated injury.

## 8. Summary

AMPAR are expressed widely by glial cells throughout the nervous system. Their activation modulates numerous cellular actions in glia including ion channel function, gene expression, migration, various aspects of growth and differentiation, and even the expression and subunit composition of AMPAR themselves. Additionally, at the tissue level glial AMPAR influence homeostatic functions and shape neuronal function by regulating myelin production and modulating synaptic function. Numerous pathological conditions, including several notable neurological and neurodegenerative conditions, involve dysregulation of extracellular glutamate levels. These conditions can lead to excessive AMPAR stimulation triggering injury responses within glia and releasing additional excitotoxic signals in the form of glutamate and cytokines. Glia are a key feature of the nervous system whose normal activities maintain an environment that is optimal for healthy development and neuronal circuit activity, thus the identification of therapeutic approaches capable of protecting glial functions and viability under excitotoxic conditions are a critical target for future research.

## Figures and Tables

**Table 1:** Summary of AMPAR expression and functions in astrocytes.

Region	GluA Expression	Ca^2+^ Permeability		Functions	
		Degree	Evidence	Physiological	Pathophysiological
Cb	GluA1, GluA4 [96]	++	ePhys [96]	Synapse modulation [40] Blockade of K^+^ currents [97]	Altered motor control [98] GLAST downregulation [99]
Ctx	GluA2 dominant (qPCR) [100]	+/−	Ci [101]	Regulate K^+^ currents [48]	Excitotoxicity [101]
Hp	GluA2 dominant (qPCR) [100] No expression (ephys) [102]	No AMPA current	ePhys [102]	N.D.	N.D.
OB	GluA1, 2, 4 (IHC) [103]	+	ci, ePhys [103]	N.D.	N.D.
Th	GluA1-4 (RT-PCR) [104]	+/−	ePhys [104]	N.D.	N.D.

+/− low Ca^2+^ permeability; + intermediate Ca^2+^ permeability; ++ high Ca^2+^ permeability Cb: cerebellum; Ci: calcium imaging analysis; ePhys: electrophysiological analysis; Ctx: cortex; Hp: hippocampus; IHC: immunohistochemistry; N.D.: not determined; OB: olfactory bulb; qPCR: quantitative real-time PCR; RT-PCR: reverse transcription PCR; Th: Thalamus.

**Table 2:** Summary of AMPAR expression and functions in oligodendrocytes.

Preparation	GluA Expression	Ca^2+^ Permeability		Functions	
		Degree	Evidence	Physiological	Pathophysiological
OPC in vitro	GluA2, 3, 4 (qPCR, WB) [154,155]	++	Ci [155,169] ePhys [155]	IEG expression [44]	Excitotoxicity [170] OGD injury [171,172,173] NF-Y-dependent apoptosis [45]
OPC ex vivo	N.D.	+	Ci, ePhys [161]	Proliferation, Differentiation [41,43] Migration [39] Blockade of K^+^ currents [43,174] OPC LTP [175]	OGD injury [165]
OPC in vivo	GluA2-4 (FISH) [46]	N.D.	N.D.	Survival [165]	H-I injury [172] Remyelination [148,176]
OL in vitro	GluA2, 3, 4 (qPCR, WB) [155]	+	Ci [155,177] ePhys [155]	N.D.	Excitotoxicity [65]
OL ex vivo	GluA4 (IHC) [106]	+	Ci [109]	Activate NMDAR [109]	Excitotoxicity [106]
OL in vivo	GluA2/3 (IHC) [177] GluA4 IHC [167]	N.D.	N.D.	N.D.	Demyelination [119] H-I injury [178] Spinal cord injury [179]

+ intermediate Ca^2+^ permeability; ++ high Ca^2+^ permeability Cb: cerebellum; Ci: calcium imaging analysis; ePhys: electrophysiological analysis; IEG: immediate early gene; IHC: immunohistochemistry; H-I: hypoxic ischemia; FISH: fluorescent in situ hybridization; N.D.: not determined; OGD: oxygen glucose deprivation; qPCR: quantitative real-time PCR; WB: Western blot.

**Table 3:** Summary of AMPAR expression and functions in microglia.

Preparation	GluA Expression	Ca^2+^ Permeability		Functions	
		Degree	Evidence	Physiological	Pathophysiological
In vitro	GluA2,3,4 (RT-PCR) [233] GluA1,2,3 (qPCR) [234] GluA1 (ICC) [236]	-	ePhys [225,226]	Chemotaxis [117]	Hypoxia induced increase in GluA2-4 [237] Release of IL-1b and TNF-a [234,237] TNF-a release [234]
In vivo	GluA2/3, 4 (IHC) [237] No AMPAR (ePhys) [238]	No AMPAR	ePhys [238]	N.D.	Hypoxia/Ischemia increased GluA2-4 [237,239]

–: negligible Ca^2+^ permeability; ePhys: electrophysiological analysis; ICC: immunocytochemistry; IHC: immunohistochemistry; qPCR: quantitative real-time PCR; RT-PCR: reverse transcription PCR; N.D.: not determined.

**Table 4:** Summary of AMPAR expression and functions in other glia.

Tissue Age	GluA Expression	Ca^2+^ Permeability		Functions	
		Degree	Evidence	Physiological	Pathophysiological
RG Developing Tissue	GluA1, 2, 3 (qPCR, ICC) [251] GluA3, 4 (qPCR) [252] GluA3,4 [252]	+	Ci [251]	N.D.	N.D.
RG Adult tissue	GluA2, 3 and 4 (qPCR) [166]	N.D.	N.D.	Proliferation, survival [253]	N.D.
Tanycte	GluA2/3 (IHC) [254]	N.D.	N.D.	N.D.	N.D.
Schwann cell	GluA2/3, 4 (IHC, I-EM) [255] Functional AMPAR in developing cells [256]	+	ePhys [256,257]	ATP release [258]	N.D.
Satellite glia	GluA4 (IHC) [259,260]	+	Ci [259,260]	N.D.	N.D.
Enteric glia	GluA1, 3 (qPCR, WB) [261]	N.D.	N.D.	N.D.	N.D.

+: intermediate Ca^2+^ permeability; ePhys: electrophysiological analysis; ICC: immunocytochemistry; I-EM: immunogold electron microscopy; IHC: immunohistochemistry; qPCR: quantitative real-time PCR; RG: Radial Glia; N.D.: not determined; Western blot: WB.
